# 1-Oxoisoindoline-2-carboxamide

**DOI:** 10.1107/S1600536808004923

**Published:** 2008-02-27

**Authors:** Bushra Maliha, Ishtiaq Hussain, M. Nawaz Tahir, Muhammad Ilyas Tariq, Hamid Latif Siddiqui

**Affiliations:** a University of the Punjab, Institute of Chemistry, Lahore 54590, Pakistan; b University of Sargodha, Department of Physics, Sargodha, Pakistan; c University of Sargodha, Department of Chemistry, Sargodha, Pakistan

## Abstract

The title mol­ecule, C_9_H_8_N_2_O_2_, is essentially planar. The crystal structure is stabilized by hydrogen bonding. An intra­molecular N—H⋯O hydrogen bond results in a six-membered ring. Each mol­ecule inter­acts with two others through N—H⋯O and C—H⋯O hydrogen bonding, resulting in the formation of nine-membered rings. These hydrogen bonds generate a two-dimensional polymeric network. There are also π–π inter­actions between the aromatic and heterocyclic rings [centroid–centroid distance 3.638 (2) Å].

## Related literature

For related literature, see: Berger *et al.* (1999 ▶); Cignarella *et al.* (1981 ▶); Goddard (1977 ▶); Goddard & Levitt (1979 ▶); Maliha *et al.* (2007 ▶); Mancilla *et al.* (2007 ▶); Momose (1980 ▶); Zuman (2004 ▶).
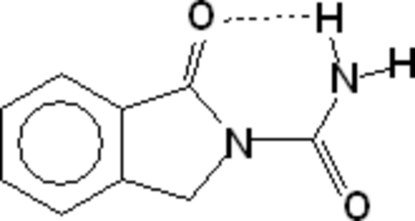


## Experimental

### 

#### Crystal data

C_9_H_8_N_2_O_2_
*M* *_r_* = 176.17
Orthorhombic, 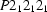

*a* = 3.9839 (3) Å
*b* = 7.8732 (8) Å
*c* = 25.651 (2) Å
*V* = 804.58 (13) Å^3^
*Z* = 4
Mo *K*α radiation
μ = 0.11 mm^−1^
*T* = 296 (2) K
0.25 × 0.12 × 0.10 mm

#### Data collection

Bruker Kappa APEXII CCD diffractometer
Absorption correction: multi-scan (*SADABS*; Bruker, 2005 ▶) *T*_min_ = 0.975, *T*_max_ = 0.990
5461 measured reflections
1254 independent reflections
860 reflections with *I* > 2σ(*I*)
*R*_int_ = 0.037

#### Refinement

*R*[*F* ^2^ > 2σ(*F* ^2^)] = 0.040
*wR*(*F* ^2^) = 0.138
*S* = 1.07
1254 reflections
124 parameters
H atoms treated by a mixture of independent and constrained refinement
Δρ_max_ = 0.23 e Å^−3^
Δρ_min_ = −0.22 e Å^−3^

### 

Data collection: *APEX2* (Bruker, 2007 ▶); cell refinement: *APEX2*; data reduction: *SAINT* (Bruker, 2007 ▶); program(s) used to solve structure: *SHELXS97* (Sheldrick, 2008 ▶); program(s) used to refine structure: *SHELXL97* (Sheldrick, 2008 ▶); molecular graphics: *ORTEP-3 for Windows* (Farrugia, 1997 ▶) and *PLATON* (Spek, 2003 ▶); software used to prepare material for publication: *WinGX* (Farrugia, 1999 ▶) and *PLATON*.

## Supplementary Material

Crystal structure: contains datablocks global, I. DOI: 10.1107/S1600536808004923/at2545sup1.cif

Structure factors: contains datablocks I. DOI: 10.1107/S1600536808004923/at2545Isup2.hkl

Additional supplementary materials:  crystallographic information; 3D view; checkCIF report

## Figures and Tables

**Table 1:** Hydrogen-bond geometry (Å, °)

*D*—H⋯*A*	*D*—H	H⋯*A*	*D*⋯*A*	*D*—H⋯*A*
N2—H2*A*⋯O1	0.95 (3)	1.91 (3)	2.710 (3)	140 (2)
N2—H2*B*⋯O2^i^	0.88 (3)	2.08 (3)	2.943 (3)	167 (3)
C8—H8*A*⋯O2^ii^	0.97	2.57	3.447 (4)	151

Symmetry codes: (i) 
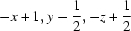
; (ii) 
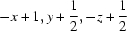
.

**Fig. 1.**
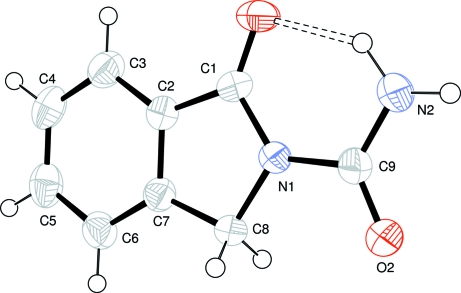
The ORTEP diagram of the title compound (I) with displacement ellipsoids at 50% probability level; intramolecular interaction has been indicated by broken line. H-atoms are shown by small circles of arbitrary radii.

**Fig. 2.**
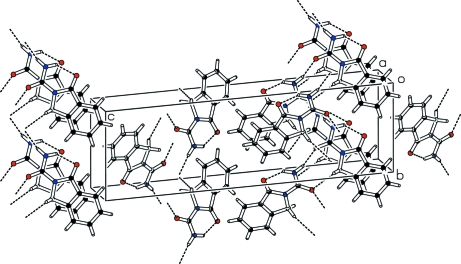
The packing figure (PLATON: Spek, 2003) which shows the H-bonding and the π-π interaction.

## supplementary crystallographic information

### Comment

A number of isoindole type compounds are known due to their wide importance in pharmaceutical industry (Berger *et al.*, 1999; Cignarella *et al.*, 1981). Several isoindoles have exhibited anti-inflammatory and analgesic activity (Mancilla *et al.*, 2007). Certain substituted isoindoles have wide applications as herbicides (Goddard, 1977; Goddard *et al.*, 1979). In continuation to our studies of *ortho*-phthaldehyde with various types of ureas (Maliha *et al.*, 2007), the present compound is isolated when simple urea is reacted as given in preparation. The estimation of urea present in the biological fluids is determined with the help of color development (Momose, 1980; Zuman, 2004) when it is reacted with *ortho*-phthaldehyde. This fact was utilized for the formation of the title compond (I).

For comparison the best molecule is of 1-oxo-*N*-phenylisoindoline-2- carboxamide (Maliha *et al.*, 2007). The bond distances in the aromatic ring (A) containing C3 are in the range of 1.379 (4) Å to 1.392 (4) Å. The formation of heterocyclic ring (B: C1/N1/C8/C7/C2) containing carbonyl group (C1?O1) and attached to ring (A), affects the bond angles in the aromatic ring. These bond angles vary in the range [118.1 (3)°-121.2 (3)°]. In this range there are three values which are compareable for diagonal atoms. The range of the bond angles in the heterocyclic ring is [1.396 (3) Å - 1.500 (4) Å], in comparison to [1.3865 (17) Å - 1.5016 (18) Å] as reported in 1-oxo-*N*-phenylisoindoline-2-carboxamide. The molecule is essentially planar with a maximum deviation of -0.028 (3) Å for N2. There exists an intramolecular H-bond [N2—H2A···O1], thus forming a six membered ring as shown in Fig 1. The O1-atom is not involved in intermolecular H-bonding. There exist intermolecular H-bond of N—H···O and C—H···O type as given in the Table 1. This kind of H-bond links each asymmetric unit at two places as shown in Fig 2. The distance between ring centroids of aromatic and heterocyclic is 3.638 (2) Å along the *a* axis, which is indication of π-π interaction.

### Experimental

A mixture of *o*-phthaldehyde (0.67 g, 200 mmol) and urea (0.30 g, 200 mmol) in 100 ml of ethanol was refluxed for 6 h. A blue color developed. The flask contents were allowed to stand for 24 h at room temperature. A white solid was separated from the solution and was washed with ethanol,ether and hexane respectively, and dried in open air. The crystals suitable for X-ray diffraction were grown in a mixture of acetone-ethanol (1:1) by slow evaporation at room temperature. The compound is soluble in DMSO, DMF, acetone, ethyl acetate, and partially soluble in ethanol and chloroform [m.p.: 493 K, yield: 55%].

### Refinement

H atoms were positioned geometrically, with C—H = 0.93, 0.97 Å for aromatic and methylene C-atoms and constrained to ride on their parent atoms. The H-atoms attached to N2 were taken from fourier synthesis and their coordinates were refined. The thermal parameter of all H-atoms was taken 1.2 times *U*_eq_ of the parent atom.

### Crystal data

**Table:**

C_9_H_8_N_2_O_2_	*F*_000_ = 368
*M**_r_* = 176.17	*D*_x_ = 1.454 Mg m^−^^3^
Orthorhombic, *P*2_1_2_1_2_1_	Mo *K*α radiation λ = 0.71073 Å
Hall symbol: P 2ac 2ab	Cell parameters from 1295 reflections
*a* = 3.9839 (3) Å	θ = 1.6–28.6º
*b* = 7.8732 (8) Å	µ = 0.11 mm^−^^1^
*c* = 25.651 (2) Å	*T* = 296 (2) K
*V* = 804.58 (13) Å^3^	Needle, colourless
*Z* = 4	0.25 × 0.12 × 0.10 mm

### Data collection

**Table:**

Bruker KappaAPEXII CCD diffractometer	1254 independent reflections
Radiation source: fine-focus sealed tube	860 reflections with *I* > 2σ(*I*)
Monochromator: graphite	*R*_int_ = 0.037
Detector resolution: 7.40 pixels mm^-1^	θ_max_ = 28.6º
*T* = 296(2) K	θ_min_ = 1.6º
ω scans	*h* = −3→5
Absorption correction: multi-scan(SADABS; Bruker, 2005)	*k* = −9→10
*T*_min_ = 0.975, *T*_max_ = 0.990	*l* = −34→22
5461 measured reflections	

### Refinement

**Table:**

Refinement on *F*^2^	Secondary atom site location: difference Fourier map
Least-squares matrix: full	Hydrogen site location: inferred from neighbouring sites
*R*[*F*^2^ > 2σ(*F*^2^)] = 0.040	H atoms treated by a mixture of independent and constrained refinement
*wR*(*F*^2^) = 0.138	*w* = 1/[σ^2^(*F*_o_^2^) + (0.0804*P*)^2^] where *P* = (*F*_o_^2^ + 2*F*_c_^2^)/3
*S* = 1.07	(Δ/σ)_max_ < 0.001
1254 reflections	Δρ_max_ = 0.23 e Å^−^^3^
124 parameters	Δρ_min_ = −0.22 e Å^−^^3^
Primary atom site location: structure-invariant direct methods	Extinction correction: none

### Special details

**Table:**

Geometry. All e.s.d.'s (except the e.s.d. in the dihedral angle between two l.s. planes) are estimated using the full covariance matrix. The cell e.s.d.'s are taken into account individually in the estimation of e.s.d.'s in distances, angles and torsion angles; correlations between e.s.d.'s in cell parameters are only used when they are defined by crystal symmetry. An approximate (isotropic) treatment of cell e.s.d.'s is used for estimating e.s.d.'s involving l.s. planes.
Refinement. Refinement of *F*^2^ against ALL reflections. The weighted *R*-factor *wR* and goodness of fit *S* are based on *F*^2^, conventional *R*-factors *R* are based on *F*, with *F* set to zero for negative *F*^2^. The threshold expression of *F*^2^ > σ(*F*^2^) is used only for calculating *R*-factors(gt) *etc*. and is not relevant to the choice of reflections for refinement. *R*-factors based on *F*^2^ are statistically about twice as large as those based on *F*, and *R*- factors based on ALL data will be even larger.

### Fractional atomic coordinates and isotropic or equivalent isotropic displacement parameters (Å^2^)

**Table:**

	*x*	*y*	*z*	*U*_iso_*/*U*_eq_	
O1	0.1443 (8)	0.5881 (3)	0.09333 (8)	0.0599 (8)	
O2	0.3962 (6)	0.7070 (2)	0.24721 (7)	0.0479 (7)	
N1	0.1909 (7)	0.7618 (2)	0.16626 (8)	0.0335 (6)	
N2	0.3940 (9)	0.4951 (3)	0.18736 (10)	0.0512 (8)	
H2A	0.346 (10)	0.476 (4)	0.1514 (13)	0.061*	
H2B	0.478 (10)	0.421 (4)	0.2092 (14)	0.061*	
C1	0.1002 (9)	0.7240 (3)	0.11498 (10)	0.0380 (7)	
C2	−0.0491 (8)	0.8806 (3)	0.09388 (10)	0.0351 (7)	
C3	−0.1770 (10)	0.9115 (4)	0.04430 (11)	0.0450 (8)	
H3	−0.1780	0.8269	0.0190	0.054*	
C4	−0.3025 (9)	1.0715 (4)	0.03378 (12)	0.0491 (8)	
H4	−0.3903	1.0952	0.0010	0.059*	
C5	−0.2985 (9)	1.1968 (4)	0.07165 (12)	0.0489 (9)	
H5	−0.3837	1.3038	0.0639	0.059*	
C6	−0.1693 (9)	1.1655 (4)	0.12114 (11)	0.0427 (7)	
H6	−0.1652	1.2504	0.1463	0.051*	
C7	−0.0474 (8)	1.0053 (3)	0.13185 (10)	0.0343 (7)	
C8	0.1037 (9)	0.9367 (3)	0.18109 (9)	0.0335 (7)	
H8A	0.3014	1.0006	0.1912	0.040*	
H8B	−0.0569	0.9384	0.2095	0.040*	
C9	0.3350 (8)	0.6532 (3)	0.20346 (10)	0.0350 (7)	

### Atomic displacement parameters (Å^2^)

**Table:**

	*U*^11^	*U*^22^	*U*^33^	*U*^12^	*U*^13^	*U*^23^
O1	0.102 (2)	0.0398 (11)	0.0383 (10)	0.0154 (14)	−0.0116 (14)	−0.0118 (9)
O2	0.0734 (18)	0.0362 (11)	0.0340 (10)	0.0018 (12)	−0.0113 (11)	0.0002 (8)
N1	0.0453 (16)	0.0263 (10)	0.0289 (10)	0.0045 (11)	−0.0024 (10)	−0.0006 (8)
N2	0.081 (2)	0.0327 (13)	0.0404 (13)	0.0173 (15)	−0.0087 (15)	0.0015 (10)
C1	0.050 (2)	0.0358 (14)	0.0286 (12)	−0.0004 (15)	−0.0013 (13)	−0.0047 (11)
C2	0.0382 (18)	0.0348 (14)	0.0323 (12)	0.0001 (13)	0.0018 (13)	0.0021 (11)
C3	0.050 (2)	0.0502 (17)	0.0345 (13)	0.0036 (18)	−0.0019 (14)	0.0011 (13)
C4	0.047 (2)	0.064 (2)	0.0366 (13)	0.0042 (19)	−0.0034 (14)	0.0140 (14)
C5	0.047 (2)	0.0477 (18)	0.0518 (17)	0.0100 (17)	0.0017 (16)	0.0159 (15)
C6	0.0473 (19)	0.0352 (14)	0.0455 (15)	0.0051 (16)	0.0040 (15)	0.0022 (12)
C7	0.0365 (18)	0.0344 (14)	0.0321 (12)	0.0019 (13)	0.0018 (12)	0.0016 (11)
C8	0.0437 (19)	0.0274 (12)	0.0293 (11)	0.0002 (14)	0.0001 (12)	−0.0027 (10)
C9	0.0408 (18)	0.0314 (13)	0.0326 (12)	−0.0014 (15)	0.0028 (13)	0.0020 (11)

### Geometric parameters (Å, °)

**Table:**

O1—C1	1.218 (3)	C3—C4	1.382 (4)
O2—C9	1.224 (3)	C3—H3	0.9300
N1—C1	1.396 (3)	C4—C5	1.385 (5)
N1—C9	1.404 (3)	C4—H4	0.9300
N1—C8	1.470 (3)	C5—C6	1.392 (4)
N2—C9	1.332 (3)	C5—H5	0.9300
N2—H2A	0.95 (3)	C6—C7	1.379 (4)
N2—H2B	0.87 (3)	C6—H6	0.9300
C1—C2	1.472 (4)	C7—C8	1.500 (4)
C2—C7	1.383 (4)	C8—H8A	0.9700
C2—C3	1.392 (4)	C8—H8B	0.9700
			
C1—N1—C9	128.0 (2)	C4—C5—C6	121.2 (3)
C1—N1—C8	112.5 (2)	C4—C5—H5	119.4
C9—N1—C8	119.4 (2)	C6—C5—H5	119.4
C9—N2—H2A	114 (2)	C7—C6—C5	118.3 (3)
C9—N2—H2B	120 (2)	C7—C6—H6	120.8
H2A—N2—H2B	126 (3)	C5—C6—H6	120.8
O1—C1—N1	125.4 (3)	C6—C7—C2	120.5 (2)
O1—C1—C2	128.8 (2)	C6—C7—C8	129.7 (2)
N1—C1—C2	105.8 (2)	C2—C7—C8	109.8 (2)
C7—C2—C3	121.4 (3)	N1—C8—C7	102.36 (19)
C7—C2—C1	109.5 (2)	N1—C8—H8A	111.3
C3—C2—C1	129.1 (2)	C7—C8—H8A	111.3
C4—C3—C2	118.1 (3)	N1—C8—H8B	111.3
C4—C3—H3	121.0	C7—C8—H8B	111.3
C2—C3—H3	121.0	H8A—C8—H8B	109.2
C3—C4—C5	120.6 (3)	O2—C9—N2	124.9 (3)
C3—C4—H4	119.7	O2—C9—N1	119.6 (2)
C5—C4—H4	119.7	N2—C9—N1	115.5 (2)
			
C9—N1—C1—O1	−2.8 (5)	C5—C6—C7—C8	−179.9 (3)
C8—N1—C1—O1	−179.9 (3)	C3—C2—C7—C6	−0.9 (5)
C9—N1—C1—C2	178.1 (3)	C1—C2—C7—C6	178.8 (3)
C8—N1—C1—C2	1.0 (3)	C3—C2—C7—C8	179.9 (3)
O1—C1—C2—C7	−179.5 (3)	C1—C2—C7—C8	−0.4 (4)
N1—C1—C2—C7	−0.4 (4)	C1—N1—C8—C7	−1.2 (3)
O1—C1—C2—C3	0.2 (6)	C9—N1—C8—C7	−178.5 (2)
N1—C1—C2—C3	179.3 (3)	C6—C7—C8—N1	−178.1 (3)
C7—C2—C3—C4	0.2 (5)	C2—C7—C8—N1	0.9 (3)
C1—C2—C3—C4	−179.5 (3)	C1—N1—C9—O2	−179.3 (3)
C2—C3—C4—C5	0.3 (5)	C8—N1—C9—O2	−2.4 (4)
C3—C4—C5—C6	−0.1 (5)	C1—N1—C9—N2	0.6 (5)
C4—C5—C6—C7	−0.6 (5)	C8—N1—C9—N2	177.5 (3)
C5—C6—C7—C2	1.1 (5)		

### Hydrogen-bond geometry (Å, °)

**Table:**

*D*—H···*A*	*D*—H	H···*A*	*D*···*A*	*D*—H···*A*
N2—H2A···O1	0.95 (3)	1.91 (3)	2.710 (3)	140 (2)
N2—H2B···O2^i^	0.88 (3)	2.08 (3)	2.943 (3)	167 (3)
C8—H8A···O2^ii^	0.97	2.57	3.447 (4)	151

Symmetry codes:  (i) −*x*+1, *y*−1/2, −*z*+1/2; (ii) −*x*+1, *y*+1/2, −*z*+1/2.
